# Mitochondrial uncoupling proteins regulate angiotensin‐converting enzyme expression: crosstalk between cellular and endocrine metabolic regulators suggested by RNA interference and genetic studies

**DOI:** 10.1002/icl3.1019

**Published:** 2015-08-02

**Authors:** Sukhbir S. Dhamrait, Cecilia Maubaret, Ulrik Pedersen‐Bjergaard, David J. Brull, Peter Gohlke, John R. Payne, Michael World, Birger Thorsteinsson, Steve E. Humphries, Hugh E. Montgomery

**Affiliations:** ^1^ Centre for Cardiovascular Genetics, BHF LaboratoriesUniversity College LondonLondonUK; ^2^ Department of CardiologyWestern Sussex Hospitals NHS TrustWest SussexUK; ^3^ Centre INSERM U897‐Epidemiologie‐BiostatistiqueBordeauxFrance; ^4^ Department of Cardiology, Nephrology and EndocrinologyHillerød HospitalHillerødDenmark; ^5^ Faculty of Health SciencesUniversity of CopenhagenCopenhagenDenmark; ^6^ Department of CardiologyThe Whittington Hospital NHS TrustLondonUK; ^7^ Institute of Experimental and Clinical PharmacologyUniversity Hospital of Schleswig‐HolsteinKielGermany; ^8^ Scottish National Advanced Heart Failure ServiceGolden Jubilee National HospitalClydebankUK; ^9^ Royal Centre for Defence MedicineQueen Elizabeth HospitalBirminghamUK; ^10^ UCL and National Centre for Sport, Exercise & HealthUniversity College LondonLondonUK; ^11^ UCL Institute for Human Health and PerformanceUniversity College LondonLondonUK

**Keywords:** ACE, association studies, endothelial cell, gene expression, genetics, uncoupling protein

## Abstract

Uncoupling proteins (UCPs) regulate mitochondrial function, and thus cellular metabolism. Angiotensin‐converting enzyme (ACE) is the central component of endocrine and local tissue renin–angiotensin systems (RAS), which also regulate diverse aspects of whole‐body metabolism and mitochondrial function (partly through altering mitochondrial UCP expression). We show that ACE expression also appears to be *regulated by* mitochondrial UCPs. In genetic analysis of two unrelated populations (*healthy young UK men* and *Scandinavian diabetic patients*) serum ACE (sACE) activity was significantly higher amongst *UCP3‐55C* (rather than T) and *UCP2* I (rather than D) allele carriers. RNA interference against UCP2 in human umbilical vein endothelial cells reduced UCP2 mRNA sixfold (*P* < 0·01) whilst increasing ACE expression within a physiological range (<1·8‐fold at 48 h; *P* < 0·01). Our findings suggest novel hypotheses. Firstly, cellular feedback regulation may occur between UCPs and ACE. Secondly, *cellular* UCP regulation of *sACE* suggests a novel means of crosstalk between (and mutual regulation of) cellular and endocrine metabolism. This might partly explain the reduced risk of developing diabetes and metabolic syndrome with RAS antagonists and offer insight into the origins of cardiovascular disease in which UCPs and ACE both play a role.

**Abbreviations**
ACE: Angiotensin‐converting enzyme
ACEI: Angiotensin‐converting enzyme inhibitor
ADP: Adenosine diphosphate
Ang I: Angiotensin I
Ang II: Angiotensin II
ANCOVA: Analysis of covariance
ANOVA: Analysis of variance
ARB: Angiotensin II type 1 receptor blocker
AT_1_R: Angiotensin II type 1 receptor
AT_2_R: Angiotensin II type 2 receptor
ATP: Adenosine triphosphate
BAT: Brown adipose tissue
cDNA: Complementary deoxyribonucleic acid
Ct: Crossing threshold
D: Deletion
DNA: Deoxyribonucleic acid
H^+^: Hydrogen ion/proton
HMAR: Healthy Male Army Recruits Study
HUVECS: Human umbilical vein endothelial cells
GAPDH: Glyceraldehyde 3‐phosphate dehydrogenase
I: Insertion
LD: Linkage disequilibrium
mRNA: Messenger ribonucleic acid
O_2_: Molecular/diatomic oxygen
PCR: Polymerase chain reaction
RAS: Renin–angiotensin system
RNA: Ribonucleic acid
ROS: Reactive oxygen species
RT‐PCR: Reverse transcription polymerase chain reaction
sACE: Serum angiotensin‐converting enzyme
SD: Standard deviation
siRNA: Small interfering ribonucleic acid molecule
SNP: Single nucleotide polymorphism
T1DM: Type 1 Diabetes Mellitus Study
UCP: Uncoupling protein

## Introduction

#### Both circulating and local (tissue/cellular) renin–angiotensin systems exist

As a pivotal component of the endocrine renin–angiotensin systems (RAS), angiotensin‐converting enzyme (ACE) plays an important role in the regulation of the human circulation. Cleaved from an anchoring stalk on endothelial cells that line blood vessels, it is released into the circulation, acting upon the protein angiotensin I to yield eight amino‐acid angiotensin II (Ang II). Ang II provokes release of the hormone aldosterone from the adrenal glands, which leads to salt and water retention by the kidney. It also causes constriction of small blood vessels in the arterial tree – actions which, together, serve to elevate blood pressure. In medical care, RAS antagonists, whether ACE inhibitors (ACEIs) or drugs antagonising the actions of Ang II at its type 1 receptor (AT_1_R blockers or ARBs), are widely used in the treatment of elevated blood pressure, or in the treatment of heart failure in which reduced vascular tone and reduced circulating volume may offer advantages.

But the RAS has functions far beyond this, being found in cells and tissues throughout the body. Here, Ang II action on cell surface AT_1_R and type 2 (AT_2_R) receptors affects the function of the cell that synthesised it (autocrine actions), or the function of nearby cells (paracrine actions). Ang II can also act as an intracellular signalling molecule (so‐called intracrine action): some cells internalise Ang II made elsewhere, and others synthesise it *de novo* (reviewed in 1). In this way, both circulating and tissue RAS can act independently or interact in the regulation of cell function.

#### ACE activity is influenced by naturally occurring variation in the *ACE* gene

Circulating and tissue ACE activity varies greatly between individuals, and common genetic variation in the *ACE* gene explains up to 40% of such differences. In particular, each of the two inherited *ACE* genes can exist in one of two forms. One form contains a small extra sequence of DNA (287 base pairs) and is known as the ‘insertion’ or ‘I’ variant (allele). If this fragment is missing, the gene variant is known as the deletion, or ‘D’, allele. In both the circulating/endocrine 2 and cellular 3 RAS, the ‘I’ allele is associated with lower ACE activity.

#### RAS play an important role in regulating metabolism in health and disease

One important function of local and endocrine RAS is in the regulation of cellular and whole‐body metabolism. This they do in numerous ways, influencing, for example, the storage and release of fatty acid fuels from fat cells (adipocytes) 4; regulating islet cells in the pancreas, which are responsible for releasing the hormone insulin and thus regulating uptake and use of glucose 5; and regulating the uptake and use of carbohydrate fuel by the liver 6. But Ang II influences more than the uptake of metabolic substrates. It increases liver, skeletal muscle and whole‐body oxygen consumption in rodents 7, 8, 9. Conversely, ARBs and ACEI reduce oxygen consumption related to renal sodium transport 10. Human data are supportive of such metabolic roles: the *ACE* I allele is associated not only with lower circulating and tissue ACE activity but also with successful physical performance in hypoxic environments 11, 12, 13, 14 and with enhanced training‐related falls in skeletal muscle oxygen consumption per unit of external work 15, 16.

These metabolic roles of RAS appear to influence the development of disease in humans. Genetically determined high ACE activity (marked by the ACE D rather than I allele) is associated with the development of metabolic syndrome (hypertension, diabetes and abnormal blood lipid profile) 17, whilst reducing RAS activity (by the use ACEIs or ARBs) also reduces the risk of people developing diabetes 18, or of them suffering a myocardial infarction (heart attack), clinical signs or symptoms of heart failure, stroke or death from a cardiovascular cause 19.

#### Ang II has direct effects on mitochondria

The *cellular* metabolic effects of RAS may be mediated, in part, by direct action of Ang II on the mitochondrial respiratory chain (reviewed in 20). Mitochondria are the intracellular organelles responsible for generating the body's energy currency, adenosine triphosphate (ATP). The respiratory or electron transport chain of the inner mitochondrial membrane consists of an assembly of several discrete electron carriers, which are grouped into complexes. Three of these complexes (complexes I, III and IV) work as oxidation–reduction‐driven proton pumps: electrons derived from diverse metabolic substrates combine with molecular oxygen to form water, and the energy released drives the translocation of protons (hydrogen ions, H^+^) from the mitochondrial matrix, across the otherwise impermeable inner membrane, and into the intermembrane space. This results in a chemiosmotic gradient (a mitochondrial membrane potential) across the inner membrane, which drives the flow of these protons back into the matrix through ATP synthase, which produces ATP from adenosine diphosphate (ADP) and inorganic phosphate. When the membrane potential is high (for instance at rest when no useful work is being performed and the demand for ATP is low), complexes I and III are also able to produce reactive oxygen species (ROS), where diatomic oxygen (O_2_) combines with a single electron only to form superoxide rather than being fully reduced to water. These ROS can cause substantial cell damage 21. Exogenously administered Ang II traffics to mitochondria 22, 23, where outer mitochondrial membranes may express AT_1_Rs 24. Ang II will then stimulate production of ROS, NADPH oxidase‐dependent superoxide and ADP‐independent respiration – which reduces the activities of complexes I and III. Mitochondria may have the capacity to endogenously synthesise Ang II 25, 26, 27, 28, 29.

#### Uncoupling proteins can ‘short circuit’ the mitochondrial membrane and reduce the membrane potential

The ‘coupling’, which connects substrate energy with the derived ATP is, however, incomplete – protons can flow back into the matrix in a manner disconnected from ATP synthesis. This is in part controlled by nuclear‐encoded, mitochondrial‐targeted uncoupling proteins (UCPs), of which five mammalian forms are recognised 30. Of these, UCP4 and UCP5 are principally neuronally expressed 31. The remaining three (UCP1–3) have close sequence homology. Expression of UCP2 is recognised in tissues including white adipose tissue, liver, and cardiac and skeletal muscle, whilst that of UCP1 is limited to brown adipose tissue (BAT), and of UCP3 largely to BAT and skeletal and, to a lesser extent, cardiac muscle 32. By allowing protons to flow back across the inner mitochondrial membrane without coupled ATP synthesis, UCPs reduce the membrane potential and help protect the cell from the generation of excessive ROS from complexes I and III 33, 34. Thus, expression of UCP2 is increased as ROS levels rise, in a negative feedback regulatory system 35. Indeed, it has been hypothesised that UCPs evolved in response to severe ROS formation resulting from the beta‐oxidation of fat 36.

#### UCPs alter metabolism through mechanisms in addition to altering mitochondrial membrane potential

This said, UCP2 may have metabolic roles other than, or beyond those related to, proton gradient and ROS generation (well reviewed in detail in 37). Indeed, it appears to increase fatty acid oxidation 38, whilst long‐chain fatty acid intake is also known to induce UCP2 expression 39. Further, UCP2 appears to play roles in the regulation of food intake 14, 15 and insulin secretion 16, 17, although the mechanisms by which such actions occur remain poorly understood 40. One means by which to explore the physiological roles of UCPs is through the study of genetic variants that are associated with differences in their expression. Helpfully, even a common genetic variation in the *UCP3*/*2* locus influences UCP expression.

#### Functional variation exists in the genes that encode the UCPs

**Figure 1:**
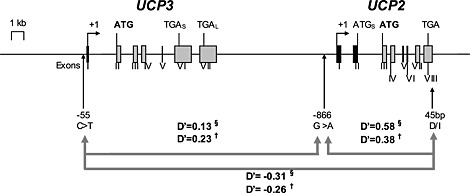
Diagrammatic representation of the human *UCP3* and *UCP2* gene locus (chromosome 11q13) with the relative positions and linkage disequilibria (LD) of the three common genetic variants in this study: the *UCP3‐55C*>*T* promoter variant, *UCP2‐866G*>*A* promoter variant and the *UCP2 D*/*I* variant. *UCP2* is approximately 7 kb downstream from *UCP3*. They share 72% sequence homology. The exonic structures are shown (rectangles) as well as the transcription start points (angled arrows), translation start codons (ATG) and translation stop codons (TGA). *UCP3* has two translation stop codons, yielding either a short (TGA_S_) or long (TGA_L_) transcript. The short transcript does not code for a functioning protein. There are three ATG codons at positions 888, 924 and 948 within an open reading frame in exon 2 of UCP2, which could initiate translation of a putative short protein (depicted as ATG_S_). However, translation only starts at the ATG codon in exon 3 to yield UCP2 protein 47. LD (or D′) is the non‐random association of alleles or genetic markers in a population and is measured between 0 and 1. When two alleles are completely randomly associated, D′ is zero. When two alleles are completely linked or associated, then D′ = 1. In the figure, D′ is shown between the three gene variants for the HMAR (§) and T1DM (†) studies. In both studies, there was significant LD between the *UCP2*‐866G>A and *UCP2 D*/*I* variants (D′ 0·38 – 0·54; *P* < 0·0001) and negative LD (−0·31 to −0·26; *P* = 0·0005) between the *UCP3*‐55C>T and *UCP2 D*/*I* variants, but no LD between the *UCP3*‐55C>T and *UCP2*‐866G>A variants. The common *UCP3*‐55C allele therefore showed significant allelic association with the rare *UCP2* I allele, and the latter with the rare *UCP2*‐866A allele.

A common variant exists in the *UCP2* gene's promoter region (the area that initiates gene transcription), comprising the presence of an adenine base (rather than the more frequent guanidine: *UCP2*‐866G>A, rs659366). This variant lies in a region containing binding sites for transcription factors (factors that stimulate gene transcription) influenced by hypoxia and inflammation. Linkage disequilibrium (LD) refers to the finding that some gene variants are not randomly distributed in a population: being in proximity, they tend to ‘travel together’. This is the case for this UCP2 variant (Fig. 1), which is in complete LD with a second promoter variant (−2723T>A). These also explain 71% of the variation in messenger RNA (mRNA) transcript ratio associated with the deletion/insertion (D/I) of 45 base pairs of DNA (in exon 8 of the gene) 41. The *UCP2‐866A* allele is associated with lower gene transcription (repression) in somatic non‐β cells 42 but more effective *UCP2* gene transcription in pancreatic β cells (those that make insulin). In humans, the *UCP2‐866A* allele is thus associated with reduced insulin sensitivity 42 and glucose‐stimulated insulin secretion 43, diabetes in obese subjects 42, oxidative stress in diabetic people 44 and future cardiovascular risk in diabetic and non‐diabetic people 44. The *UCP2* I allele is associated with reduced *UCP2* mRNA stability 41, and with elevated body mass index 45 and variably with basal metabolic rate 46.

Meanwhile, a common variant in the regulatory ‘promoter’ region of the *UCP3* gene (the presence of a thymidine base rather than the more frequent cytosine: −55C>T, rs1800849) is in negative LD with the *UCP2* D/I polymorphism 48, the T allele being associated with obesity in a recessive manner 48 and with higher circulating low‐density lipoproteins cholesterol levels 49. For clarity, such LD is demonstrated in Fig. 1, which relates to our study populations described later. We have recently shown the combined presence of both the *UCP3‐55C allele* and the *UCP2‐866A* allele (the ‘*UCP3*‐55C/*UCP2*‐866A haplotype’) to be associated with gains in muscle performance following endurance training 50 in a similar pattern to that previously associated with the *ACE* I allele (lower ACE activity) 51.

#### ACE may regulate UCP expression. But do UCPs regulate ACE expression?

Some of the metabolic effects associated with ACE and Ang II (refer to the previous discussion) are now thought to be mediated through regulation of UCP expression: Ang II upregulates expression of UCP1 52, UCP2 53 and UCP3 54. However, an unexpected (and completely novel) finding made by our group suggested an inverse relationship: that UCP activity might also regulate ACE expression. We had previously reported a correlation between lower serum ACE (sACE) activity and decreased risk of hypoglycaemia in Danish diabetic adult, going on to show a similar association of the *ACE* I allele (i.e. genetically determined low sACE) with a lower risk of severe hypoglycaemia 55. Low ACE activity is favourable for performance and hypoglycaemia awareness when substrate availability is limited, for example, during hypoglycaemia. As part of our ongoing work, we recently genotyped these subjects for the *UCP3*‐55C>T polymorphism, finding an association between *UCP3* genotype and sACE activity. Such data suggest a role for UCPs in the regulation of ACE activity, rather than the (previously recognised) converse observation.

We sought to confirm the validity of the finding by performing more detailed genetic analysis in this sample of 210 type 1 diabetic study patients (≥18 years old; 117 women; mean age 45·5 ± 13·7 years; none taking RAS antagonists): the *T1DM* study. We then sought replication of our findings in a second completely independent dataset, comprising 250 Caucasian British healthy male army recruits (mean age 19·4 ± 2·2 years): the *HMAR* study.

Finally, as sACE is derived from endothelial cells, we performed *in vitro* experimentation to confirm the co‐expression of both *ACE* and *UCP2* mRNA in human umbilical vein endothelial cells (HUVECs) before determining whether changes in *UCP* expression (by RNA interference) could directly alter endothelial *ACE* expression.

## Results

#### Gene variants in the adjacent UCP2 and UCP3 loci are tightly associated

Genotype distributions (Table 1) were in Hardy–Weinberg equilibrium (meaning that they are consistent with stability over generations) and similar to those previously reported in Caucasians 41, 45, 48. There was significant LD between the *UCP2*‐866G>A and *UCP2* D/I variants (D′ 0·38–0·54; *P* < 0·0001; Fig. 1) and negative LD (−0·31 to −0·26; *P* = 0·0005) between the *UCP3*‐55C>T and *UCP2* D/I variants, as previously described in a South Indian cohort 48, but no LD between the *UCP3*‐55C>T and *UCP2*‐866G>A variants. The *UCP3*‐55C allele therefore showed allelic association with the rare *UCP2* I allele, and the latter with the *UCP2*‐866A allele. There was no association between any genotype and common descriptive population variables.

**Table 1:** *UCP3‐55C*>*T*, *UCP2‐866G*>*A* and *UCP2 D*/*I* genotype distributions and rare allele frequencies (with 95% confidence intervals in parentheses) in the HMAR and T1DM studies

**Study**	**Gene variant**		
	***UCP3‐55C*>*T***	***UCP2‐866G*>*A***	***UCP2 D*/*I***
HMAR	128/71/13	82/108/22	104/92/16
	0.229 (0.189–0.269)	0.358 (0.313–0.404)	0.292 (0.249–0.336)
T1DM	116/78/16	84/98/28	116/76/18
	0.262 (0.220–0.304)	0.367 (0.321–0.413)	0.267 (0.224–0.309)
*P* HMAR vs. T1DM	0.53	0.97	0.74

There was no significant difference in genotype distribution between the two studies.

#### sACE activity is strongly associated with *UCP2/3* genotype

sACE activity was normally distributed in both studies and weakly correlated with age (HMAR *r* = −0·19; *P* = 0·02, T1DM *r* = 0·12; *P* = 0·04) but no other population variables. Age‐adjusted sACE was therefore used in subsequent analyses. As expected, sACE activity was higher in T1DM subjects, with levels similar to those of previously published data 56. The wide distribution of ACE activities in the T1DM group may partly reflect differences in diabetic phenotype, such as the presence or absence of nephropathy 56.

**Figure 2:**
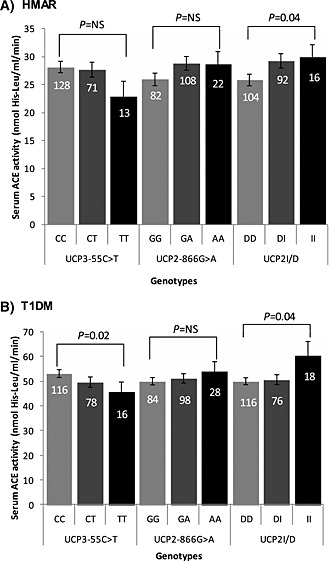
Mean age‐adjusted serum ACE (sACE) activity by *UCP3‐55C*>*T*, *UCP2‐866G*>*A* and *UCP2 D*/*I* genotypes in **A**: healthy male army recruits (HMAR) study and **B**: type 1 diabetic people (T1DM) study. Data are mean ± SD. The number of individuals in each genotype group is given at the top of each bar. The *P* value for the statistical comparison of mean age‐adjusted sACE activity between genotypes is shown above each gene variant. There were significant differences in sACE activity by *UCP2D*/*I* genotype in both studies and by *UCP3‐55C*>*T* genotypes in the T1DM study. NS = non‐significant.

In both studies, sACE activity differed by *UCP* genotype. For *UCP2*, the I allele was associated with significantly higher sACE activity in both the HMAR study (25·8 ± 8·3 vs. 29·2 ± 10·3 vs. 29·9 ± 7·3 nmol his‐leu/ml/min for *DD* vs. *DI* vs. *II*, linear trend *P* = 0·04; I allele vs. DD homozygotes *P* = 0·02) and T1DM study (49·9 ± 15·5 vs. 50·5 ± 17·6 vs. 60·4 ± 24·2 nmol his‐leu/ml/min; *P* = 0·04 analysis of variance [ANOVA]). Considering *UCP3*, the ACE activity for *CC* versus *CT* versus *TT* genotypes was 53·0 ± 17·0 versus 49·5 ± 18·3 versus 45·5 ± 16·6 nmol his‐leu/ml/min (*P* = 0·02 ANOVA) in the T1DM cohort. In the HMAR group, ACE activity was 28·1 ± 9·6 versus 27·7 ± 8·4 versus 22·8 ± 8·7 nmol his‐leu/ml/min (*P* = 0·07 ANOVA), with sACE activity lower amongst *UCP3TT* homozygotes in both cohorts (by, on average, 6–8 nmol his‐leu/ml/min; Fig. 2), a finding confirmed on repeated measures analysis in the HMAR study (*P* = 0·03). In multivariate analysis, the *statistical interaction* between the three *UCP* genotypes accounted for up to 4·1% and 5% of the inter‐individual variation in sACE activity in the HMAR and T1DM studies, respectively, but with the majority of this variation accounted for by the statistical interaction between *UCP2*‐866G>A and *UCP2*D/I genotypes (*P* = 0·001).

**Figure 3:**
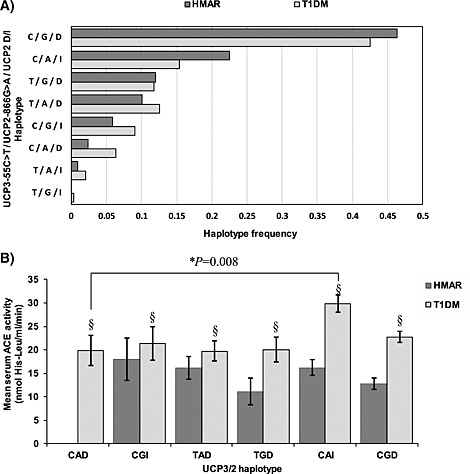
**A**: The *UCP3*‐55C>T, *UCP2*‐866G>A and *UCP2*D/I haplotype frequencies are shown in descending order of frequency for both the HMAR and T1DM studies. The most common haplotype is the *UCP3‐55C*/*UCP2‐866G*/*UCP2D* (*CGD*) *allele*. Subsequent analysis was confined to common haplotypes with a frequency greater than 0.05. **B**: Predicted *UCP3*‐55C>T, *UCP2*‐866G>A and *UCP2*D/I haplotype effects on age‐adjusted serum ACE (sACE) activity in the HMAR and T1DM study subjects. Data are mean ± SD for each single allele effect. Haplotypes with a frequency less than 0.05 are not represented in the figure because of the very small group size and resulting wide standard deviation for sACE activity. §There was a significant haplotype effect in predicting sACE activity overall in the T1DM subjects (*P* = 0·01), but not in the HMAR study. *The bracket shows the comparison in the T1DM study between those individuals with the *CA**D*** haplotype who had the lowest mean sACE activity and those with the *CA**I*** haplotype who had the highest mean sACE activity. A SNP change from the *UCP3*/*2 CA**D*** to *CA**I*** haplotype led to a significant mean increase in sACE activity of 10·0 nmol his‐leu/ml/min in the T1DM study (*P* = 0·008). Individuals with the *CAD* haplotype in the HMAR study also had the lowest sACE activity but are not shown owing to the low sample size.

The expected sACE activity phenotypic means according to estimated haplotypes (groupings of the different gene variants, found in combination) for study subjects in the HMAR and T1DM studies are depicted in Fig. 3. Uncommon haplotype groups with a frequency <0.05 in both studies were excluded from subsequent analysis (i.e. the *UCP3*‐55T/*UCP2*‐866A/*UCP2*I and the *UCP3*‐55T/*UCP2*‐866G/*UCP2*I haplotypes). There was insufficient statistical power to detect a haplotypic effect in predicting sACE activity in the HMAR study (*P* = 0·24), perhaps contributed to by the limited number in such haplotype groups and compounded by the different distribution on ACE activity observed in this group when compared with T1DM subjects (see section on Discussion). However, there was a significant haplotype effect in predicting sACE activity in the T1DM subjects (*P* = 0·01). The lowest sACE activity was seen amongst individuals with the *UCP3*/*2* haplotype *CAD* in both studies. A single nucleotide polymorphism (SNP) change from the *UCP3*/*2* CA**D** to CA**I** haplotype led to a significant mean increase in sACE activity of 10·0 nmol his‐leu/ml/min in the T1DM study (*P* = 0·008). The *UCP2* I allele has been associated with decreased mRNA stability, with expression dependent on variation in the *UCP2G*>*A* polymorphism 41. Thus, genotypes associated with putative lower UCP2 expression were associated with *higher* sACE activity.

#### The association of *UCP2/3* genotype with sACE activity might in part be mediated through UCP's regulation of ACE expression

In theory, differences in UCP expression may drive systemic (whole body) physiological effects (such as changes in insulin secretion, or in inflammatory or immune responses), which might change sACE activity in ways yet unknown. Alternatively, changes in cellular UCP expression might more directly influence ACE expression in those same cells. We sought to clarify the latter hypothesis through *in vitro* experimentation. Specifically, we sought to determine whether cellular *ACE* gene expression (ACE mRNA levels) would alter when UCP expression (*UCP* mRNA activity) was reduced by introducing targeted ‘small interfering RNA’ molecules (siRNA). We performed this experiment in HUVECs, given that circulating ACE is derived from such vascular endothelial cells.

**Figure 4:**
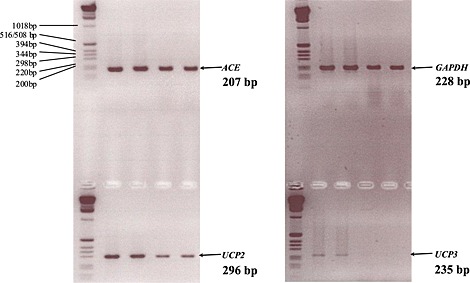
Agarose gel electrophoresis confirmed that *UCP2*, *UCP3* and *ACE* mRNA are co‐expressed in endothelial cells. Non‐quantitative RT‐PCR for *GAPDH*, *UCP2*, *ACE* and *UCP3* was performed on mRNA isolated from four separate cultures of HUVECs grown to confluency, and the products run on a 2% agarose gel against a 1 kb DNA ladder. There is less product in the last two lanes, but this does not affect the result.

Firstly, the mRNA co‐expression of *ACE*, *UCP2*, *UCP3* and *GAPDH* (a housekeeping gene) in HUVECs was confirmed using a two‐step reverse transcription polymerase chain reaction amplification (RT‐PCR). Both *UCP2* and *ACE* mRNA were found to be expressed abundantly in cultured HUVECs, whilst expression of *UCP3* was confirmed at a lower level (Fig. 4).

**Figure 5:**
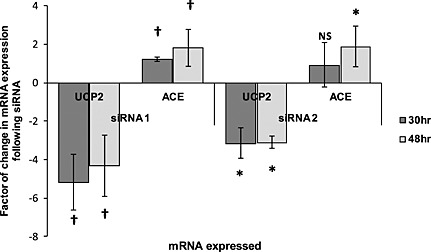
UCP2 RNA interference results in a significant increase in ACE mRNA. HUVECs were transfected with two different siRNA against UCP2 (siRNA 1 and siRNA 2) and total RNA isolated at 30 and 48 h. Quantitative RT‐PCR was performed on an ABI prism 7900HT. UCP2 RNA interference resulted in a significant decrease between threefold and sixfold in UCP2 mRNA expression at 30 and 48 h associated with a significant increase in ACE mRNA (1.2‐ to 1.4‐fold at 30 h and 1.8‐fold at 48 h). (Experiments were repeated a minimum of five times; data are mean ± SD.) †*P* < 0.0001. *P < 0.001.

Subsequent quantitative PCR confirmed that *UCP2* mRNA expression was reduced at all time points (between threefold and sixfold, *P* < 0·0001, Fig. 5) in endothelial cells transfected with two different siRNAs against *UCP2* (siRNA1 and siRNA2), when compared with scrambled siRNA (a negative control, not specifically targeted at UCP2). In keeping with data from our genetic study, this effect was associated with a significant *increase* in *ACE* expression, *ACE* mRNA rising at 30 and 48 h (1·2‐ to 1·4‐fold and approximately 1·8‐fold, respectively, *P* < 0·001).

## Discussion

#### Genetic variation in the UCP3/2 locus is associated with sACE activity

Ours is the first study to demonstrate an association of sACE with a genetic variant outside the *ACE* gene itself 57, 58, 59. We showed that the *UCP3‐55*C (rather than T) and *UCP2* I (rather than D) alleles were associated with higher age‐adjusted sACE activity, the interaction between the three genotypes accounting for 4·1% and 5%, respectively, of the inter‐individual variation in age‐adjusted sACE activity in two independent genotyping studies – one of healthy young UK Caucasian men and one of Scandinavian middle‐aged diabetic people. The lowest sACE was associated with *UCP3*‐55C/*UCP2*‐866A/*UCP2*D (*UCP3*/*2* CAD) haplotype in both studies, an SNP change from *UCP3*/*2* CA**D** to CA**I** increasing sACE by 10·0 nmol his‐leu/ml/min in the T1DM. We are unable to confidently attribute the observed effects to a precise allele, given that the *UCP3*‐55C allele showed allelic association with the rare *UCP2* I allele, and the latter with the *UCP2*‐866A allele. Even the haplotypes described may simply mark functional variation elsewhere in the cluster. The use of exome sequencing in substantially expanded cohorts is thus suggested.

The replication of the association of UCP genotype with sACE activity in two independent and diverse groups strengthens confidence in the validity of the findings. It is worthy to note that ACE activity was substantially higher in the T1DM group than in HMAR group – a finding unlikely related to the use of two different methodologies of ACE assay. Indeed, an established literature shows ACE activity (when assayed using the same commercial *Sigma* assay we used) to be elevated in diabetic people to levels commensurate with those we identified and with similar levels in controls to those we report 56. The use of two different methodologies relates to the different geographical locations of sample acquisition and analysis. Both techniques are considered reliable and are well validated. A small systematic bias, related to the assay used, would not in any way affect the findings or conclusions of our study.

#### UCP2 RNA interference increases ACE mRNA expression

Most importantly, however, our data suggest that this association may partly result from UCP2's modulation of *ACE* expression. We confirmed the expression of UCP2 reported in endothelial cells of diverse origin 60, 61, 62, 63. Those of the vasculature are central to the pathophysiology of atherosclerosis. Indeed, *UCP2* genotype is associated with the development of vascular disease 44, whilst ACE inhibition appears protective 64.

In HUVECs, a threefold to sixfold reduction in *UCP2* mRNA (induced by transfection with two separate and different siRNA against *UCP2*) was reproducibly associated with an increase in *ACE* mRNA expression (1·2–1·4‐fold at 30 h, and a near doubling at 48 h, *P* < 0·01) – the scale of altered *ACE* expression (1·2–1·8‐fold) by UCP2 RNA interference being similar to that observed in the physiological context 65, 66, 67, 68. We cannot discount an additional influence on ACE stalk cleavage, however, and would advocate further investigation of this possibility.

#### Is there negative feedback between UCP2 and ACE at the cellular level?

UCPs regulate mitochondrial metabolism. Mitochondrial regulation by RAS 7, 9, 20, 25, 69 may partly be mediated through modulation of UCP expression: Ang II upregulates expression of tissue UCP1 in brown fat 52 and UCP2 expression in brain 70, pancreatic beta‐cells 53, 71, and hepatic 72, renal 73 and cardiac 74 tissue. ACE inhibition reduces UCP2 expression in adipose and cardiac tissue 68, 75, whilst adipose UCP3 expression is reduced in AT_2_R knockout rodents 54. However, Ang II's influence on UCP expression may be both tissue and context dependent, given the existence of seemingly conflicting data: in other studies, Ang II action at the AT_1_R *reduces* cardiac UCP2 and UCP3 expression 76; adipose UCP1 expression is *elevated* in *AT_1_R* gene knockout mice 77 and those treated with AT_1_R antagonists 78; whilst ACE inhibition may have no effect on cardiac/skeletal muscle UCP3 upregulation in response to thyroid hormones 79 or may actually increase UCP2 expression in retinal tissue 80.

Meanwhile, RAS influences whole‐body metabolism in other ways (e.g. through altered adipocyte function 4 and hepatic metabolism 6). Given this, our data suggest several hypotheses. Firstly, ACE‐UCPs feedback control may exist: this would function by Ang II altering mitochondrial function through changes in UCP expression, the latter feeding back to change Ang II synthesis through regulation of ACE expression. Secondly, UCPs might be considered to regulate mitochondrial metabolism through changes in coupling, but also through independent mitochondrial effects of Ang II. Such hypotheses would be supported were UCP expression is shown to regulate that of ACE in cells beyond those derived from the endothelium.

#### The regulation of endocrine sACE by cellular UCP may represent a novel interface between local cellular metabolism and endocrine metabolic regulation

UCP2 negatively regulates both pancreatic β cell glucose‐stimulated insulin secretion 81 and central neuronal glucose control of peripheral glucose utilisation 82. Fatty acids induce both the expression and activity of UCP2 in pancreatic β cells 83, 84, and superoxide activation of UCP2 is dependent on the presence of fatty acids 35. Skeletal muscle UCP2 and UCP3 mRNA expression is induced by a high fat diet 85, and plasma free fatty acid levels are positively correlated with cardiac UCP2 protein levels 66. Starvation leads to the depletion of glycogen stores and an increased reliance on energy transduction from fatty acid oxidation. A similar period of starvation reduces sACE activity 86 and also increases UCP expression in skeletal muscle 65 – possibly as a protective mechanism against oxidative stress 35. Furthermore, lower ACE activity in HUVECs promotes a survival advantage during starvation 87. Perhaps of greater relevance, RAS antagonists prevent diabetes and improve insulin sensitivity (reviewed in 18), whilst the RAS appears to play a role in the pathogenesis of the ‘metabolic syndrome’ (hypertension/impaired glucose tolerance/dyslipidaemia) 17, 88, 89. Indeed, increased RAS activity is common in obesity and is considered a possible link with associated diabetes and hypertension 89. In this regard, elevated RAS activity in adipocytes may play a particular role 90, raising the prospect that the recognised association between *UCP2*/*3* genotype and obesity/diabetes 41, 42, 45, 46, 49, 91, 92 might be partly adipose‐RAS mediated. This does not discount, of course, a role for elevated RAS activity of endothelial (and UCP regulated) origin also playing a role. However, we would advocate further experiments to explore the influence of adipocyte UCP expression on that of ACE.

Similarly, *UCP3‐55C*/*UCP2‐866A* haplotype is associated with training‐related gains in muscle performance 50, and it is tempting to speculate that some of this effect might be ACE mediated, given the existence of similar associations with *ACE* genotype 51.

### Future directions

Whilst confident in the validity of the observations we have made, the implications remain speculative. Further studies are also required to explore the means by which UCP might alter ACE expression: is it direct or mediated, for instance, via altered ROS activity? Inhibition of UCP2 or UCP3 could induce ROS and thereby increase ACE activity. Might UCPs additionally alter sACE activity through ACE stalk cleavage and thus increase release into the circulation? In such regards, real‐time PCR analysis of the expression of other RAS components might be performed in HUVECs, transfected in the manner that we have described. RAS activity can prove protective to blood vessels (especially, perhaps, through activation of AT_2_R rather than AT_1_R) – and UCP's role in regulating such vasoprotective effects is worthy of exploration (given that reduced ROS activity would also be vasoprotective). Recent data also suggest a role for mitochondrial‐associated membrane RAS in regulating mitochondrial function – and the study of ACE activity in such regions in response to altered UCP expression might also be suggested. Exome sequencing may help identify the precise functional *UCP2*/*3* variant affecting sACE activity. Experimentation should also be extended to other cell types.

## Materials and methods

### Genetic studies

The association of *UCP3*/*2* genotype with sACE activity was explored in two distinct cohorts of European Caucasians: young healthy British men and adult Danish patients with type 1 diabetes. Ethics committee approval was obtained, as was written informed consent from all participants, with study protocols adhering to the Declaration of Helsinki (Defence Medical Services Clinical Research Committee at the Army Training Regiment, Bassingbourn, UK, and Hillerød Hospital, Hillerød, Denmark).

#### Subjects

The HMAR study sample consisted of 250 consecutive healthy male Caucasian British Army recruits (mean age 19·4 ± 2·2 years), as previously described 93, 212 of whom had complete genotypic data.

The T1DM patients study comprised 262 consecutive Danish adults (≥18 years old; 117 women; mean age 45·5 ± 13·7 years) with a >2 year history of T1DM, as previously described 94. Analysis was restricted to the 210 with complete genotype data who were both ACEI and ARB naïve.

Genomic DNA was extracted from 10 ml EDTA venous blood. Serum was aspirated from centrifuged citrated blood and stored at −20°C for subsequent blinded analysis of sACE.

#### Measurement of sACE activity

For the HMAR study, sACE was assayed with a modified fluorometric method using carbobenzoxy‐phenyl‐alanyl‐histidyl‐leucine (Z‐phe‐his‐leu) as a substrate 95. Interassay and intraassay coefficients of variation were 13% and 11%, respectively. For the T1DM study, sACE was assessed using a commercial kinetic assay (Sigma Diagnostics, St Louis, MO, USA) based on the hydrolysis of the synthetic tripeptide *N*‐[3‐(2‐furyl)acrylolyl]‐l‐phenylalanylglycylglycine to furylacryloylphenylalanine and glycylglycine. ACE activity was determined by comparing sample reaction rate with that derived from an ACE reference. The use of two different methodologies relates to the different geographical locations of sample acquisition and analysis. The observed difference in mean sACE activity between two studies does not seem to be due to differences in the assays employed.

#### Genotyping

*UCP3*‐55C>T, *UCP2*‐866G>A and UCP2*D*/*I g*enotypes were determined by PCR amplification using published primers and conditions 41, 45, 48. Products were resolved on a 7·5% polyacrylamide gel and confirmed by two independent technicians blind to all subject data, with discrepancies resolved by repeat genotyping.

### Influence of reducing UCP mRNA levels on cellular ACE expression

HUVECs (European Collection of Cell Cultures, Salisbury, UK) were cultured to 80% subconfluence before being cultured in serum‐free EGM™ for 48 h to confluence prior to all experiments.

#### siRNA transfection

Transfection was carried out according to the manufacturer's protocol. *ACE* (105310 and 103987) and *UCP2* (120334 and 120336) siRNA as well as positive (*GAPDH*, 4390849) and negative (4611) controls were purchased from Ambion (Applied Biosystems, Austin, TX, USA). siGLO Green control was purchased from Dharmacon (Lafayette, CO, USA). Briefly, cells were cultured in six‐well plates until confluent. In optiMEM‐1 (Invitrogen, Life Technologies Ltd, Paisley, UK), 200 nM siRNA (with or without the same quantity of siGLO siRNA) was diluted, with a final volume of 180 µl. Oligofectamine 10 µl (Invitrogen, Life Technologies Ltd, Paisley, UK) and 10 µl optiMEM‐1 were premixed. Both mixtures were incubated for 10 min, then mixed together, and incubated for a further 25 min. The cells were washed with optiMEM‐1 and covered with 800 µl fresh optiMEM. To each well, 200 µl of prepared mix siRNA + oligofectamine was added dropwise. The cells were incubated for 4 h at 36°C, after which a further 500 µl 30% foetal calf serum/optiMEM‐1 was added. Total RNAs were extracted 24, 30 and 48 h after transfection.

#### RNA extraction, reverse transcription and RT‐PCR

Cellular RNA was isolated using the RNeasy® Mini Kit (Qiagen, Crawley, UK) spin protocol. The RNA was stored at −80°C and quantified using a nanodrop spectrophotometer (ND‐8000, Labtech, East Sussex, UK) prior to reverse transcription. Resultant complementary DNA samples were stored at −20°C.

Human *ACE*, *UCP2*, *UCP3* and *GAPDH* (housekeeping gene) target sequences were amplified using forward and reverse primers (Table 2) by PCR to confirm co‐expression. Amplification products were separated by electrophoresis on a 2% agarose gel against a 1 kb DNA ladder.

**Table 2:** Forward and reverse primers used during non‐quantitative RT‐PCR of human *ACE*, *UCP3* and *UCP2* and the housekeeping gene *GAPDH* and the resulting amplicon size

**Gene**	**cDNA accession no.**	**Primers**	**Amplicon size, bp**
h*GAPDH*	J04038	F: GGGGAAGGTGAAGGTCGGAGT	228
		R: CCTGGAAGATGGTGATGGGAT	
h*UCP3*	U84763/AF050113	F: CCTCACTACCCGGATT	235
		R: GTTGACGATAGCATTCCT	
h*UCP2*	NM_003355/AF019409	F: GCTTTGAAGAACGGGAC	296
		R: CTGTAACCGGACTTTAGCA	
h*ACE*	J04144	F: ACCAATGACACGGAAAG	207
		R: GTGGGTTTCGTTTCGG	

RNA was extracted at 24, 30 and 48 h after HUVECs were transfected with two different siRNAs against UCP2 (siRNA1 and siRNA2). Quantitative RT‐PCR was performed on ABI prism 7900HT. Assays Hs00174179_m1, Hs00163349_m1 and Hs01106052_m1 were used in combination to TaqMan gene expression master mix to amplify *ACE*, *UCP2* and *UCP3* mRNA, respectively. Three housekeeping genes were used: *UBC* (Hs00824723_m1), *β actin* (Hs99999903_m1) and *GAPDH* (Hs99999905_m1). We did not analyse for changes in expression of an unrelated gene to check for unintentional global translational repression by interferon activation by siRNA treatment. However, we did confirm that the expression of the three housekeeping genes, *UBC*, *β actin* and *GAPDH*, did not show a significant difference upon siRNA treatment of UCP2, by comparing each of the housekeeping genes with each other; that is, comparing the UCP2 siRNA versus no treatment cells, there was no significant difference in GAPDH mRNA levels using UBC as the housekeeping control, and similarly with other pairwise comparisons. Each reaction was repeated three times in parallel and for each condition. Crossing thresholds (Ct) were obtained using relative quantification application on the SDS2·1 software (Applied Biosystems, Life Technologies Ltd, Paisley, UK). Any value that was not consistent with the rest of the triplicate was suppressed before analysis. As a positive control using GAPDH siRNA, GAPDH mRNA was down‐regulated by a factor of 5.48 (*P* = 0.001). The Ct values included in statistical analysis were the means of triplicates. Experiments were repeated between three and five times.

### Statistical analysis

Allele frequencies were estimated by gene counting. A *χ* ^2^ test was used to compare the observed numbers of each genotype with those expected for a population in Hardy–Weinberg equilibrium. LD between sites in pairwise combination was estimated 96. One‐way analysis of covariance (ANCOVA) tested for confounders. Univariate and multivariate analyses were used to measure significance of association. Differences in descriptive population variables and sACE were compared between genotype groups. For the whole sample, characteristics were compared between genotype groups (including those defined by the presence/absence of a specific allele) using one‐way ANOVA, two‐tailed unpaired *t*‐tests, linear trend analysis and one‐way ANCOVA with sex as a covariate, using either raw or log‐transformed values as appropriate. Genotype association with sACE activity in the HMAR study was compared between genotype groups and allele groups using two‐way ANOVA with repeated measures on one factor (time). All data were analysed using SPSS (SPSS Inc., IBM Corporation, USA) and Microsoft® Office Excel® 2007 (Microsoft Corporation 2006). Data are presented as means ± standard deviation unless otherwise stated. *P* values of <0·05 were considered statistically significant.
